# TLR4 signals in B lymphocytes are transduced via the B cell antigen receptor and SYK

**DOI:** 10.1084/jem.20161117

**Published:** 2017-03-29

**Authors:** Edina Schweighoffer, Josquin Nys, Lesley Vanes, Nicholas Smithers, Victor L.J. Tybulewicz

**Affiliations:** 1 The Francis Crick Institute, London NW1 1AT, England, UK; 2 Epinova DPU, Immuno-Inflammation Therapy Area Unit, GlaxoSmithKline, Stevenage SG1 2NY, England, UK; 3 Imperial College London, London W12 0NN, England, UK

## Abstract

Toll-like receptors (TLRs) play an important role in immune responses to pathogens by transducing signals in innate immune cells in response to microbial products. TLRs are also expressed on B cells, and TLR signaling in B cells contributes to antibody-mediated immunity and autoimmunity. The SYK tyrosine kinase is essential for signaling from the B cell antigen receptor (BCR), and thus for antibody responses. Surprisingly, we find that it is also required for B cell survival, proliferation, and cytokine secretion in response to signaling through several TLRs. We show that treatment of B cells with lipopolysaccharide, the ligand for TLR4, results in SYK activation and that this is dependent on the BCR. Furthermore, we show that B cells lacking the BCR are also defective in TLR-induced B cell activation. Our results demonstrate that TLR4 signals through two distinct pathways, one via the BCR leading to activation of SYK, ERK, and AKT and the other through MYD88 leading to activation of NF-κB.

## Teaser

Schweighoffer et al. demonstrate that in B cells, TLR4 transduces signals through two distinct pathways: one via the BCR to the activation of SYK, ERK, and AKT and the other via MYD88 to the activation of NF-κB.

## Introduction

B cells form a key component of the adaptive immune response. Binding of antigen to the B cell antigen receptor (BCR), surface-bound immunoglobulin, triggers intracellular signaling pathways that can lead to B cell activation. For T-dependent antibody responses, B cells receive further signals from T cells; cytokines secreted by T cells act on B cells, and CD40 ligand (CD40L) on the T cell surface transduces signals through CD40 on B cells. Together with BCR signals, these result in activation and proliferation of B cells and subsequent differentiation into germinal center B cells, memory B cells, and antibody-secreting plasma cells. In addition, B cells are able to respond to microbial products through TLRs. In vitro stimulation of B cells through TLRs results in proliferation and differentiation into antibody-secreting cells. In vivo, TLR signals contribute to T-independent antibody responses to bacteria (Alugupalli et al., 2007; Barr et al., 2009; Neves et al., 2010; Rawlings et al., 2012). The role of TLR signals in T-dependent antibody responses has been more controversial, with some studies finding that TLR signaling is dispensable (Gavin et al., 2006; Meyer-Bahlburg et al., 2007; DeFranco et al., 2012; Rawlings et al., 2012) and others finding it important for a full response (Pasare and Medzhitov, 2005; Hou et al., 2011). It is likely that the requirement for TLR signals depends on the precise context in which TLR ligands and protein antigen are presented to B cells.

The SYK tyrosine kinase plays a critical role in B cell development and function, largely because of its role in transducing signals from the BCR and the related pre-BCR (Mócsai et al., 2010). The BCR is associated with Igα (CD79A) and Igβ (CD79B) transmembrane proteins. Binding of antigen to the BCR results in phosphorylation of tandem tyrosines within the immunoreceptor tyrosine-based activation motifs (ITAMs) in the cytoplasmic domains of Igα and Igβ, by either SYK or SRC-family kinases such as LYN (Reth and Brummer, 2004). SYK binds to these phosphorylated tyrosines through its tandem SH2 domains, leading to activation of its enzymatic activity, phosphorylation of several substrates, and signal transduction to multiple pathways (Mócsai et al., 2010). Inactivation of *Syk* results in a partial block in B cell development at the pro–B cell to pre–B cell transition and a complete block at the transition from immature to mature B cells, where signals from the pre-BCR and BCR, respectively, are required for developmental progression (Cheng et al., 1995; Turner et al., 1995, 1997). Conditional deletion of *Syk* has allowed study of the role of this key kinase in mature B cells. Those studies showed that SYK is required to transduce signals from the BCR that lead to activation of B cells, and hence for antibody responses to T-dependent and -independent polysaccharide antigens (Ackermann et al., 2015). SYK is also required for survival of mature B cells, since it transduces signals from the cytokine receptor BAFFR (Schweighoffer et al., 2013). Interestingly, binding of BAFF to BAFFR leads to activation of SYK, dependent on the BCR, suggesting close cooperation between the two receptors, although this interpretation has been challenged (Hobeika et al., 2015).

Mouse B cells express several TLRs, including TLR1, TLR2, TLR3, TLR4, TLR7, and TLR9. All of these except TLR3 signal through the MYD88 adapter protein. TLR3 uses the TRIF adapter protein, and TLR4, the receptor for LPS, signals via both MYD88 and TRIF. These adapters in turn transduce signals to the activation of IκB kinase (IKK) complex, leading to NF-κB and ERK activation (Newton and Dixit, 2012). Although most studies on TLR signaling have focused on the role of these adapter proteins, some have found that SYK may also be involved in signaling from TLR4. SYK-deficient macrophages were found to have increased cytokine release in response to LPS, a TLR4 ligand, suggesting that SYK is a negative regulator of TLR4 signaling (Hamerman et al., 2005). Conversely, other studies have found that minimally oxidized LDL, another TLR4 ligand, induces SYK binding to TLR4 in macrophages, and that SYK subsequently transduces signals leading to cytokine secretion (Miller et al., 2012). However, there are no comparable studies in B cells, and it is unknown whether SYK participates in signaling from TLR4 or other TLRs in B cells.

Here we show that SYK is required for B cell activation in response to signaling through multiple TLRs. SYK transduces TLR4 signals leading to the activation of ERK and AKT, but not NF-κB signaling pathways. Furthermore, signaling from TLR4 leads to SYK phosphorylation, which is dependent on the BCR, implying that TLR4 transduces signals via the BCR to SYK that are required for B cell activation.

## Results and discussion

### SYK is required for TLR-induced B cell activation

**Figure 1.**
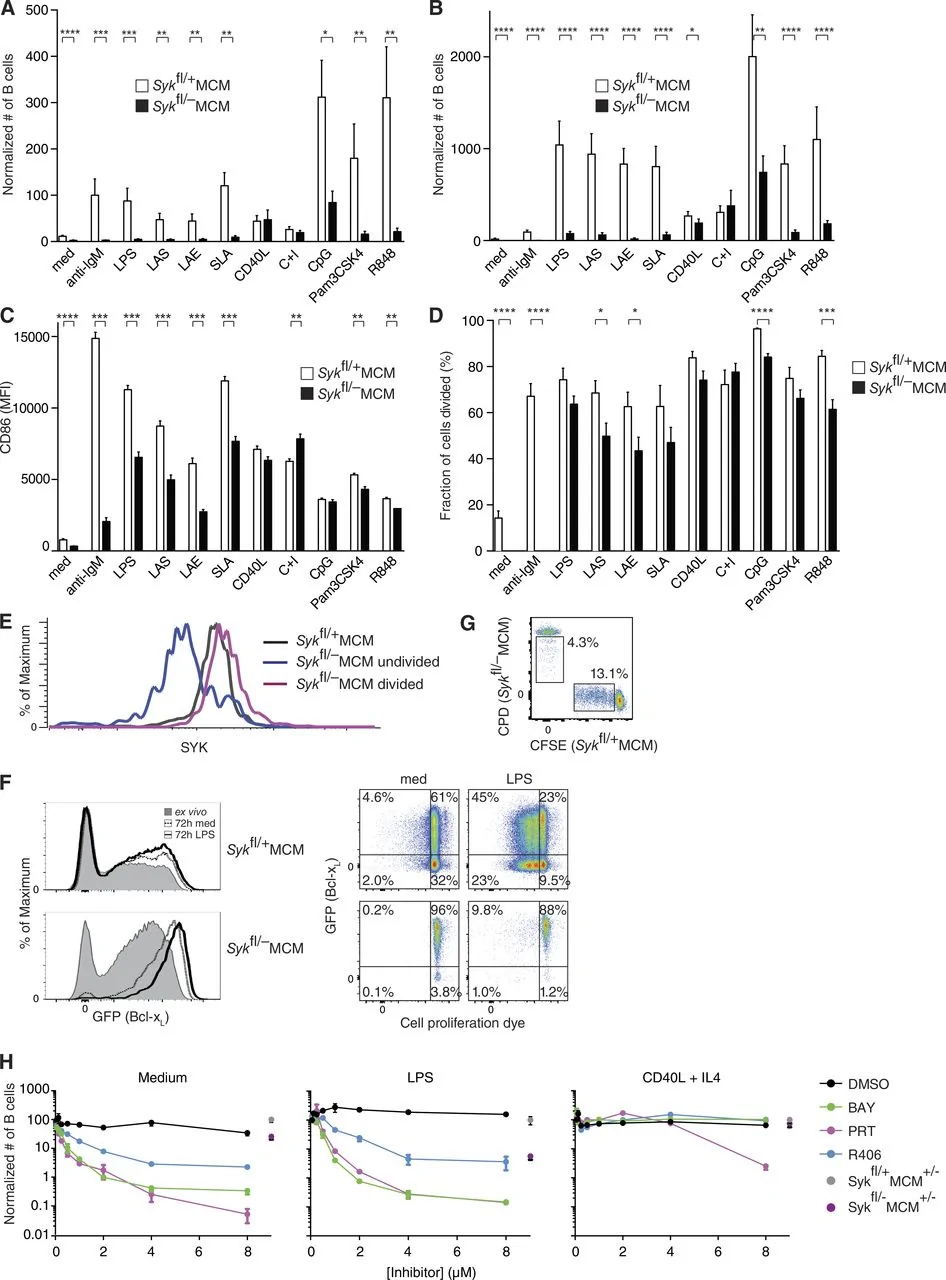
**SYK is required for TLR-induced B cell activation.** (A and B) Number of live control (*Syk^fl/+^*MCM) and mutant (*Syk^fl/−^*MCM) B cells after 48-h (A) or 72-h (B) culture with the indicated stimuli, normalized to the number in control B cells stimulated with anti-IgM, which was set to 100. Medium only (med), lipid A from *S. minnesota* (LAS) or *E. coli* (LAE), synthetic lipid A (SLA), and CD40L and IL4 (C+I). A, *n* = 6 (*Syk^fl/+^*MCM) and 8 (*Syk^fl/−^*MCM); B, *n* = 10 (*Syk^fl/+^*MCM) and 14 (*Syk^fl/−^*MCM). (C) Cell surface expression of CD86 (as measured by mean fluorescence intensity [MFI]) on control or SYK-deficient B cells cultured for 24 h with the indicated stimuli; one of three independent experiments. *n* = 8 (*Syk^fl/+^*MCM) and 6 (*Syk^fl/−^*MCM). (D) Fraction of B cells that had divided after 72 h of culture with the indicated stimuli. *n* = 10 (*Syk^fl/+^*MCM) and 14 (*Syk^fl/−^*MCM). (E) Histogram of expression of intracellular SYK measured by flow cytometry in control B cells or mutant B cells that had divided or remained undivided in cultures with LPS; representative of experiments with 11 *Syk^fl/+^*MCM and 12 *Syk^fl/−^*MCM mice. (F) B cells were isolated from radiation chimeras reconstituted with BM cells of the indicated genotypes infected with a retroviral vector expressing GFP and Bcl-x_L_. Histograms on the left show overlay of GFP expression in B cells taken straight out of mice (ex vivo) or after culture for 72 h with LPS or in medium only (med), indicating an increase in GFP expression in cultured *Syk^fl/−^*MCM B cells. 2D dot plots on the right show dilution of the CPD plotted against GFP expression, showing LPS-induced proliferation in *Syk^fl/+^*MCM but not *Syk^fl/−^*MCM B cells; numbers indicate percentage of cells in each quadrant; representative of three experiments (total *n* = 10 of each genotype). (G) Control or SYK-deficient B cells labeled with CFSE or CPD, respectively, were injected into TLR4-deficient mice that were treated with LPS. Flow cytometric analysis of CFSE^+^ or CPD^+^Syk^−^ B cells isolated from the spleen of recipient mice 3 d later shows that some B cells had undergone division as seen by dilution of dye (boxes). Percentages of cells that had divided are indicated; representative of two experiments. (H) Number of live B cells remaining after 48-h culture of wild-type B cells with a range of concentrations of three SYK inhibitors, BAY 61-3606 (BAY), PRT062607 (PRT), or R406, or with concentrations of vehicle (DMSO) equivalent to those used for the inhibitors. Cells were cultured in medium only or with LPS or CD40L + IL4, and cell numbers were normalized to cells treated with no inhibitor, which was set to 100. Representative of three independent experiments. Graph shows mean ± SEM of triplicates. For comparison, the numbers of control (*Syk^fl/+^*MCM, *n* = 6) and mutant (*Syk^fl/−^*MCM, *n* = 8) B cells remaining after 48 h of culture are also shown, normalized to the number of control cells, which was set to 100% (data taken from experiment in A). Graphs show mean ± SEM. Statistical analysis was performed using a Mann–Whitney test: *, P < 0.05; **, P < 0.01; ***, P < 0.001; ****, P < 0.0001.

To investigate a potential role for SYK in TLR signaling, we obtained SYK-deficient B cells from a mouse strain containing a loxP-flanked allele of *Syk* and a tamoxifen-inducible Cre recombinase that had been treated with tamoxifen 10 d earlier (Schweighoffer et al., 2013). We had previously shown that these B cells express no SYK, but otherwise closely resemble follicular B cells. We stimulated SYK-deficient or control mouse B cells with LPS, CpG, Pam3CSK4, and R848 (ligands for TLR4, TLR9, TLR1/TLR2, and TLR7, respectively) and measured cell survival, up-regulation of activation markers, and cell division. Strikingly, SYK-deficient B cells were strongly defective in survival in response to all four ligands (Fig. 1, A and B). In contrast, up-regulation of CD86 was partially reduced in response to LPS, Pam3CSK4, and R848 but normal in response to CpG (Fig. 1 C). Analysis of cell division, gated on the small number of surviving B cells, showed near-normal percentages of cells that had divided in response to all four TLR ligands (Fig. 1 D). However, flow cytometric analysis showed that all of these dividing cells expressed SYK, despite efficient deletion of *Syk* in the starting population of B cells (Fig. 1 E). These SYK-expressing cells are rare cells that have escaped deletion, and hence the results show there is strong selection against SYK-deficient B cells among the dividing cells, indicating that SYK may be required for TLR-induced cell division. Nonetheless, the failure to detect dividing SYK-deficient B cells could also be a consequence of their defective survival. To address this, we used a retroviral vector to ectopically express the pro-survival Bcl-x_L_ protein in both control and SYK-deficient B cells and again measured their ability to divide in response to LPS. Infected Bcl-x_L_–expressing B cells were identified by expression of GFP from the retroviral vector. After culture with LPS, we observed a strong skewing toward cells with high levels of GFP and, by inference, high levels of Bcl-xL, especially in SYK-deficient B cells (Fig. 1 F), indicating that the expression of Bcl-xL was conferring a survival advantage. Despite this improved survival, GFP^+^ SYK-deficient B cells were strongly defective in LPS-induced proliferation (Fig. 1 F), demonstrating that SYK is required for both TLR4-induced survival and proliferation.

We also stimulated the cells through the BCR with anti-IgM and with CD40L alone or with IL4. As expected, in response to anti-IgM, SYK-deficient B cells were very defective in cell survival and up-regulation of CD86 (Fig. 1, A–C); we were unable to measure cell division because there were too few surviving cells left in the cultures (Fig. 1 D). In contrast, in response to CD40L ± IL4, SYK-deficient B cells showed normal survival, division, and up-regulation of CD86. Collectively, these results show that SYK is required for TLR- but not CD40L-induced B cell activation.

Because the defect in SYK-deficient B cells was strongest in response to LPS, we decided to focus on this response, which is known to be TLR4 dependent (Hoshino et al., 1999). To extend the in vitro studies, we examined the requirement for SYK in TLR4-induced B cell responses in vivo. SYK-deficient or control B cells were injected into TLR4-deficient mice, to limit LPS responses to the transferred cells, and then the mice were challenged with LPS. Once again, we found that SYK-deficient B cells proliferated poorly compared with control cells (Fig. 1 G). Thus SYK is required for TLR4-induced B cell proliferation both in vitro and in vivo.

Furthermore, we investigated whether the kinase activity of SYK was required for TLR4 signaling, by treating wild-type B cells with three different inhibitors of SYK. All three strongly inhibited LPS-induced B cell survival, whereas the response to CD40L + IL4 was unaffected, similar to the phenotype of SYK-deficient B cells (Fig. 1 H). Thus SYK kinase activity is required for LPS-induced survival.

### SYK is required for TLR4-induced cytokine secretion

**Figure 2.**
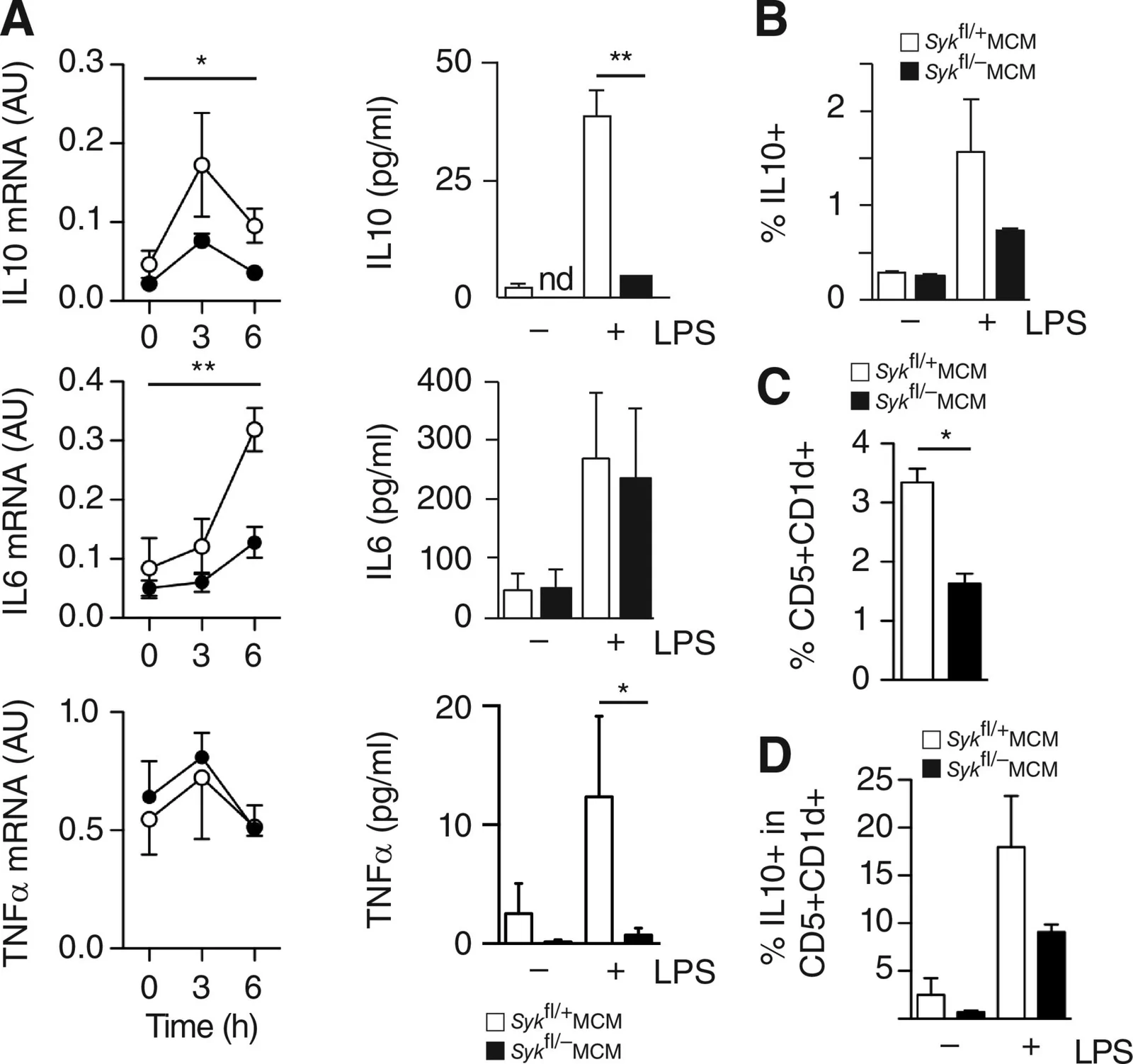
**SYK is required for TLR4-induced cytokine secretion.** (A) Levels of mRNA for IL10, IL6, and TNF in control and mutant B cells cultured for the indicated times with LPS or of cytokines secreted by B cells cultured for 16 h in the absence (−) or presence (+) of LPS. Cytokine mRNA levels were normalized to levels of *Hprt* mRNA. AU, arbitrary units; nd, not detectable. (B) Fraction of B cells positive for intracellular IL10 after culture for 5 h in the presence (+) or absence (−) of LPS. (C) Fraction of splenic B cells that were CD5^+^CD1d^+^. (D) Fraction of CD5^+^CD1d^+^ B cells positive for intracellular IL10 after culture for 5 h in the presence (+) or absence (−) of LPS. Graphs show mean ± SEM. Sample numbers: A, 3 (mRNA), 4 (cytokine protein, *Syk^fl/+^*MCM), and 6 (*Syk^fl/−^*MCM); B, 5; C, 4; and D, 5. Statistical analysis was performed using two-way ANOVA (p-values for effect of genotype; A, mRNA) and a Mann–Whitney test (A, cytokine protein; C): *, P < 0.05; **, P < 0.01.

In addition to proliferation, stimulation of B cells with TLR ligands leads to secretion of cytokines (Shen and Fillatreau, 2015). Thus we examined the potential role of SYK in this process. In response to LPS, SYK-deficient B cells were strongly defective in the secretion of IL10 and TNF, but IL6 secretion was unaffected (Fig. 2 A). Analysis of transcripts showed that in the absence of SYK, TLR4-induced IL10 and IL6 mRNA were both reduced, whereas TNF mRNA was unaffected. Thus SYK selectively regulates the secretion of some but not all cytokines, regulating IL10 and TNF production at a transcriptional and posttranscriptional level, respectively. IL10 is produced mainly by a subset of B cells characterized by the expression of CD1d and CD5 (Yanaba et al., 2008). The reduced production of IL10 could be caused by fewer IL10-producing cells or lower IL10 production. We found that in the absence of SYK there were fewer IL10-producing cells, fewer CD1d^+^CD5^+^ cells, and fewer IL10-producing cells among the latter subset (Fig. 2, B–D). Thus SYK is required for both the differentiation of B cells into CD1d^+^CD5^+^ cells and IL10 production by these cells.

### SYK transduces TLR4 signals leading to activation of ERK and AKT but not NF-κB

**Figure 3.**
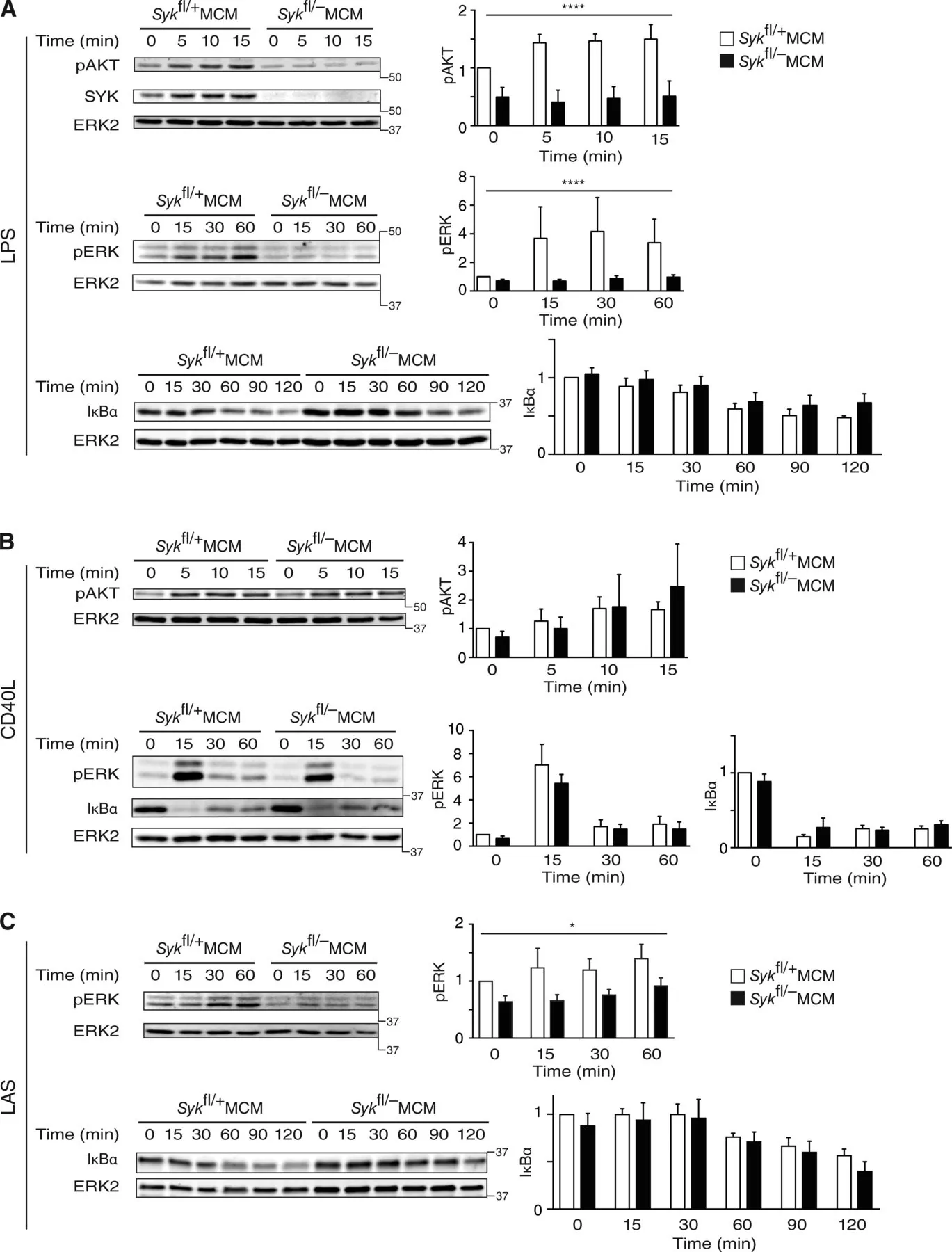
**SYK transduces TLR4 signals leading to activation of ERK and AKT but not NF-κB.** (A–C) Immunoblots of lysates from control and mutant B cells stimulated with LPS (A), CD40L (B), or LAS (C) for the indicated times. Blots were probed with antibodies to phospho-Ser473 AKT (pAKT), phospho-Thr202/Tyr204 ERK1/ERK2 (pERK), SYK, IκBα, and ERK2. Graphs show mean ± SEM levels of pAKT, pERK, and IκBα normalized to ERK2 and then to the levels in unstimulated control B cells (time 0), quantitated from multiple immunoblots. Minimum number of repeats per time point: A, 3 (pAKT), 9 (pERK), and 3 (IκBα); B, 3 (pAKT), 7 (pERK), and 6 (IκBα); C, 4 (pERK) and 4 (IκBα). Numbers on righthand edge of all blots indicate molecular mass makers (kilodaltons). Statistical analysis was performed using two-way ANOVA (p-values for effect of genotype): *, P < 0.05; ****, P < 0.0001.

To gain a better understanding of SYK’s role in TLR4 signaling, we examined the activation of ERK, AKT, and NF-κB in SYK-deficient B cells in response to LPS. We found that in the absence of SYK, LPS-induced phosphorylation of ERK and AKT was strongly reduced, whereas the degradation of IκBα, a process required for NF-κB activation, was unaffected (Fig. 3 A). In contrast, CD40L-induced activation of the same three pathways was unaffected by the loss of SYK (Fig. 3 B).

### BCR is required for TLR4-induced activation of SYK

**Figure 4.**
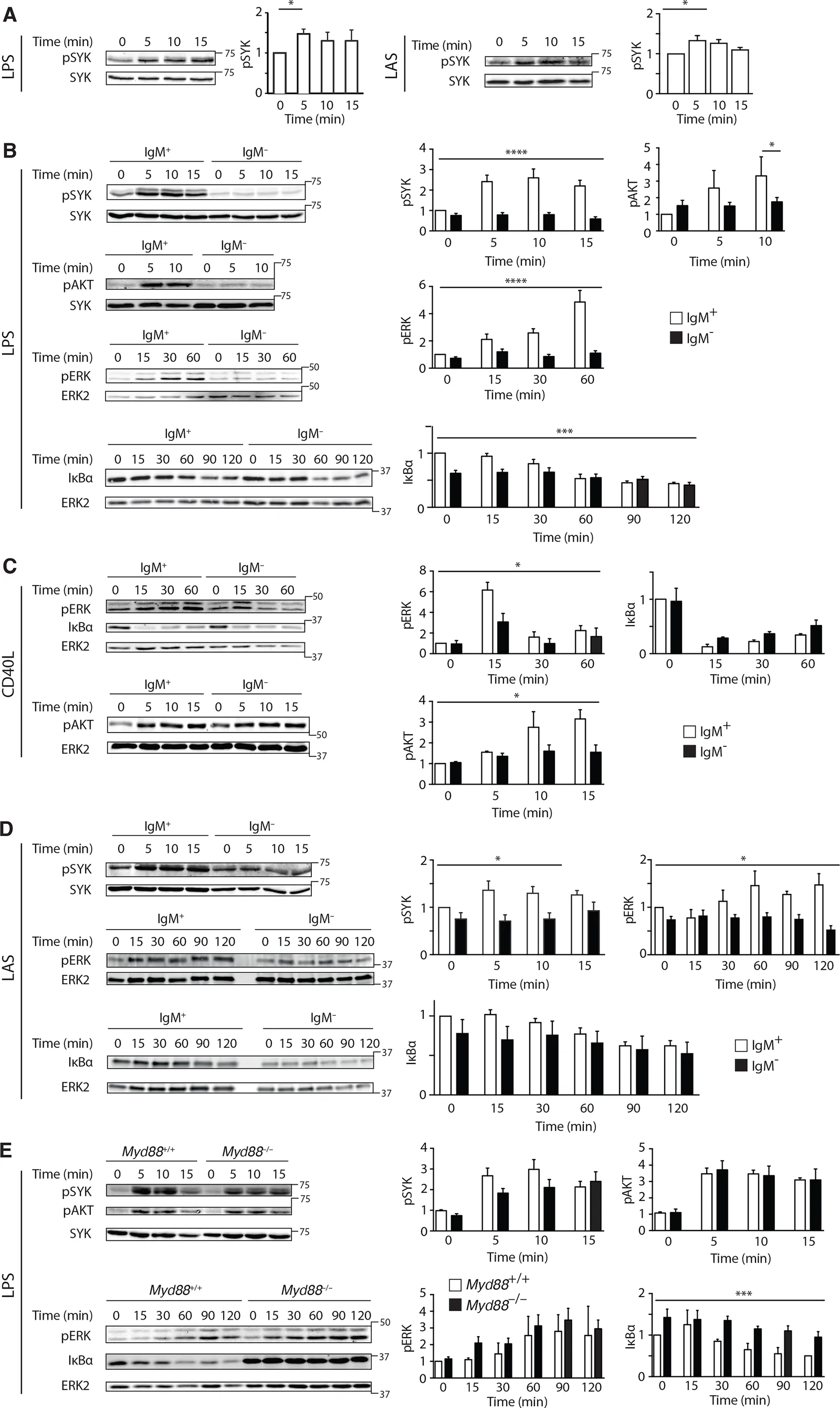
**BCR is required for TLR4-induced activation of SYK.** (A) Immunoblot of lysates from wild-type B cells stimulated for various times with LPS (left) or LAS (right). Blots were probed with antibodies to pSYK and SYK. Graphs show mean ± SEM levels of pSYK normalized to SYK or tubulin and then to unstimulated cells (time 0), quantitated from multiple immunoblots. *n* = 4 (LPS) and 3 (LAS). (B–E) Immunoblots of lysates from IgM^+^ or IgM^−^ B cells (B–D) or *Myd88^+/+^* or *Myd88^−/−^* B cells (E) stimulated with LPS (B and E), CD40L (C), or LAS (D) for the indicated times. Blots were probed with antibodies to pSYK, pAKT, pERK, SYK, ERK2, and IκBα. Graphs show mean ± SEM levels of pSYK, pAKT, pERK, and IκBα normalized to SYK or ERK2, and then to the levels in unstimulated control B cells (time 0), quantitated from multiple immunoblots. Minimum number of repeats per time point: B, 9 (pSYK except 3 for 15 min), 8 (pAKT), 7 (pERK), and 5 (IκBα); C, 3 (pERK), 3 (IκBα), and 2 (pAKT); D, 3 (pSYK), 4 (pERK), and 4 (IκBα); E, 9 (pSYK) and 7 (pAKT); 2 (pERK and IκBα in WT), 4 (pERK and IκBα in *Myd88^−/−^*). Numbers on righthand edge of all blots indicate molecular mass makers (kilodaltons). Statistical analysis was performed using a Mann–Whitney test (A and B, pAKT) or two-way ANOVA (all other graphs; p-values for effect of genotype): *, P < 0.05; ***, P < 0.001; ****, P < 0.0001.

In view of the defects in TLR4 signaling pathways in SYK-deficient B cells, we investigated whether SYK is directly activated after engagement of the receptor. We found that LPS treatment of B cells results in rapid phosphorylation of SYK, a hallmark of its activation, peaking at 5–10 min after stimulation (Fig. 4 A). In all cases where SYK is activated, a receptor with ITAM-bearing subunits is involved (Mócsai et al., 2010). In B cells, the most obvious such receptor is the BCR; hence, we tested whether the BCR is required for LPS-induced SYK phosphorylation. We made use of a mouse strain with an allele of the IgH locus containing a loxP-flanked rearranged VDJ_H_ region located upstream of the Cμ gene (*Igh^B1-8f^*; Lam et al., 1997) crossed to a tamoxifen-inducible Cre allele, and to a transgene expressing Bcl-x_L_. Treatment of these mice with tamoxifen results in loss of the BCR (both IgM and IgD) from the surface of some of the B cells, which were then separated into cells that expressed or did not express the BCR. Expression of the Bcl-x_L_ transgene allows the cells to survive despite losing expression of the BCR. Treatment of these cells with LPS showed that LPS-induced phosphorylation of SYK was lost in BCR-deficient cells (Fig. 4 B), despite normal surface levels of TLR4 (not depicted), demonstrating that the BCR is required for TLR4-induced SYK activation.

We extended this analysis to other LPS-induced pathways, and found that BCR-deficient B cells were defective in LPS-induced phosphorylation of ERK and AKT but showed largely normal degradation of IκBα (Fig. 4 B), similar to the phenotype seen in SYK-deficient cells, except that IgM^−^ cells had lower resting levels of IκBα. In contrast, BCR-deficient B cells showed partially reduced CD40L-induced phosphorylation of ERK and AKT and normal IκBα degradation (Fig. 4 C).

### BCR is required for TLR-induced B cell activation

**Figure 5.**
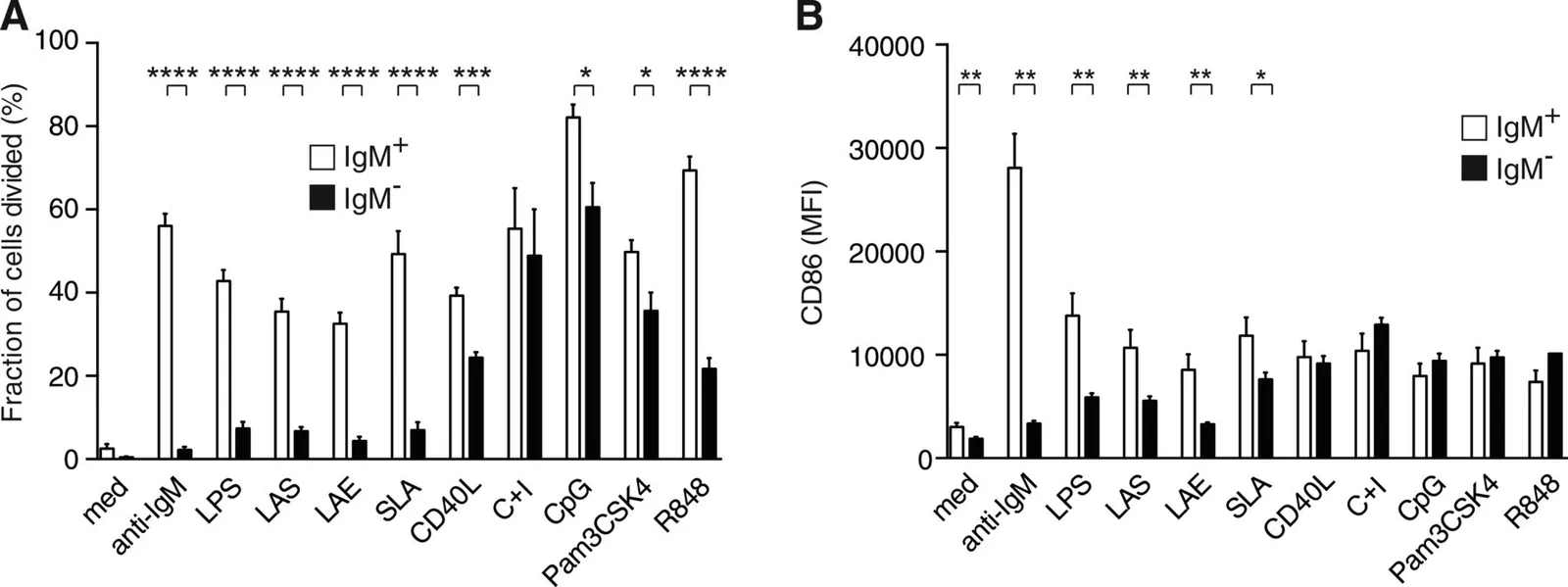
**BCR is required for TLR-induced B cell activation.** (A) Fraction of control (IgM^+^) and mutant (IgM^−^) B cells that had divided after 72 h of culture with the indicated stimuli; abbreviations of stimuli as in Fig. 1 A. Plots show combined results from three experiments with at least nine replicates for each sample, except for IgM^−^ cells stimulated with CD40L alone, for which there were six replicates. (B) Cell surface expression of CD86 (mean fluorescence intensity [MFI]) on IgM^+^ or IgM^−^ B cells cultured for 24 h with the indicated stimuli; one of two independent experiments with *n* = 5 and 6 replicates for IgM^+^ and IgM^−^ cells, respectively. Graphs show mean ± SEM. Statistical analysis was performed using a Mann–Whitney test; *, P < 0.05; **, P < 0.01; ***, P < 0.001; ****, P < 0.0001.

Because the BCR is required for TLR4-induced SYK activation, we predicted that BCR-deficient B cells would be defective in activation induced by TLR4 and potentially other TLRs. Indeed, we found that BCR-deficient B cells had strongly reduced proliferation in response to LPS and partially reduced proliferation in response to CpG, Pam3CSK4, and R848 and, as expected, were unresponsive to stimulation through the BCR (Fig. 5 A). We were unable to measure TLR-induced survival in these cells because of the ectopic expression of the pro-survival Bcl-x_L_ protein. In contrast, BCR-deficient B cells showed a partial reduction proliferation in response to CD40L and no defect in response to CD40L and IL4 (Fig. 5 A). Furthermore, BCR-deficient B cells were defective in up-regulation of CD86 in response to LPS and anti-IgM, but not in response to CpG, Pam3CSK4, R848, CD40L, or CD40L + IL4 (Fig. 5 B). Collectively, these results show that the BCR is required for B cell activation through TLRs but is largely dispensable for activation through CD40, a phenotype that is strikingly similar to that seen in SYK-deficient cells.

### Both SYK and BCR are required for the activation of B cells by lipid A

LPS is a glycolipid composed of lipid A and a repetitive polysaccharide. Binding of LPS to TLR4 and its coreceptor MD-2 is largely mediated by lipid A (Park et al., 2009). In contrast, it has been suggested that the polysaccharide part of LPS can bind to the BCR, thereby bridging the two receptors, and potentially explaining their synergistic signaling (Pone et al., 2012). Such a mode of binding could explain how LPS induces SYK phosphorylation via the BCR. To test whether this might be the case, we stimulated control and SYK-deficient B cells with lipid A alone, using lipid A from *Escherichia coli* or *Salmonella minnesota*, or, alternatively, using synthetic lipid A to eliminate any possible contaminating LPS. We found that all three forms of lipid A induced B cell survival and proliferation and up-regulation of CD86, which was defective in the absence of SYK (Fig. 1, A–D). Furthermore, BCR-deficient B cells were also defective in activation in response to lipid A (Fig. 5, A and B). We extended this analysis to lipid A–induced signaling pathways. As with LPS, lipid A induced phosphorylation of SYK in B cells (Fig. 4 A). Furthermore, lipid A–induced SYK phosphorylation was abrogated in BCR-deficient B cells, and ERK phosphorylation was reduced in the absence of either SYK or BCR, whereas lipid A–induced IκBα degradation was not dependent on either SYK or BCR (Figs. 3 C and 4 D). Collectively, these results show that the lipid A portion of LPS requires both BCR and SYK for B cell activation and demonstrate that the direct binding of LPS to the BCR is not the reason for the dependence of TLR4 signaling on BCR and SYK.

### TLR4 transduces signals to SYK phosphorylation independently of MYD88

It has been shown that MYD88 is required for TLR4-induced B cell proliferation and up-regulation of CD86 (Yanagibashi et al., 2015), both of which are SYK and BCR dependent, suggesting that MYD88 may transduce TLR4 signals to BCR and SYK. To test this hypothesis, we analyzed signaling pathways in MYD88-deficient B cells. Surprisingly, we found that LPS-induced phosphorylation of SYK, ERK, and AKT was unaffected by loss of MYD88, whereas degradation of IκB was reduced (Fig. 4 E). This demonstrates that TLR4 signals through two independent pathways, one via MYD88 leading to activation of NF-κB, and another via the BCR leading to activation of SYK, ERK, and AKT.

In summary, our results show that TLR4 transduces signals via the BCR leading to activation of SYK, and this may explain the previously observed synergy between BCR and TLR signaling in B cells leading to increased activation (Minguet et al., 2008; Pone et al., 2012). Notably, our results show that SYK plays a positive role in transducing signals from TLR4 and other TLRs, in contrast to the negative role reported in macrophages (Hamerman et al., 2005). A recent study reported that the BCR was required for B cell proliferation in response to LPS, CpG, and anti-CD40 (Otipoby et al., 2015), in agreement with our findings, except that we see only a partial defect in CD40L-induced proliferation and no defect in response to CD40L + IL4. That study proposed that the BCR contributes to CpG-induced proliferation by activating AKT, which in turn phosphorylates and inactivates GSK3β. Our work takes this further by showing a connection between TLR4 and the BCR, with the latter required to transduce TLR4 signals leading to activation of SYK, AKT, and ERK. Our results may also explain the requirement for the BTK kinase, phospholipase Cγ2, and the BLNK adapter protein in LPS-induced B cell proliferation (Khan et al., 1995; Jumaa et al., 1999; Wang et al., 2000) because all of these proteins have been shown to transduce signals from the BCR.

It has been proposed that CpG-induced signaling through TLR9 in B cells leads to SYK activation via MYD88, PYK2, and DOCK8 (Jabara et al., 2012). This is distinct from our results, which show that MYD88 is not required for LPS-induced SYK activation, suggesting that TLR4 and TLR9 may activate SYK by different mechanisms. TLR4-induced ERK activation has been shown to depend on IKK-mediated phosphorylation of p105 (NF-κB1), leading to its degradation and hence release of the TPL2 kinase, which is constitutively associated with p105, and subsequent TPL2-mediated phosphorylation and activation of MEK1, which in turn activates ERK1 and ERK2 (Banerjee et al., 2006; Gerondakis et al., 2007; Gantke et al., 2012). Combined with our results, this suggests that SYK is likely required for TLR4-induced IKK-mediated phosphorylation of p105. However, we also found that SYK is not required for TLR4-induced degradation of IκB, an event that is triggered by IKK-mediated phosphorylation of IκB. This implies that there may be two distinct pools of IKK activated downstream of TLR4: one activated via SYK leading to p105 phosphorylation and eventually ERK activation, and another activated via MYD88 leading to IκB degradation. This will be an interesting area of future study.

It is not clear how TLR4 signaling could lead to BCR-dependent SYK activation. A recent study has shown that TLR4 signaling induces increased actin dynamics, leading to lower spatial confinement of the BCR, greater mobility of the receptor, and enhanced signaling (Freeman et al., 2015). Such a mechanism could explain the connection between the two receptors, though we note that the study used B cells that had been treated with LPS overnight, whereas we detected LPS-induced SYK activation within 5 min. Finally, we note that despite transducing signals via the BCR to the activation of SYK, stimulation of B cells through TLR4 does not result in a calcium flux, in contrast to direct stimulation of the BCR (unpublished data), highlighting qualitative differences in the way TLR4 and the BCR signal despite using common signal-transducing proteins.

In conclusion, our results show that the BCR and SYK directly participate in signaling from TLR4. This is a very similar arrangement to that seen with BAFFR signaling, which also leads to BCR-dependent SYK activation (Schweighoffer et al., 2013), suggesting that this form of receptor cooperation may be a common feature of signal transduction pathways.

## Materials and methods

### Mice

Mice carrying a loxP-flanked allele of *Syk* (*Syk^tm1.1Nns^*, *Syk^fl^*), a constitutive disruption of *Syk* (*Syk^tm1Tyb^*, *Syk^−^*), and an allele of ROSA26 expressing the MerCreMer tamoxifen-inducible Cre fusion protein (Gt(ROSA)26Sor^tm1(cre/Esr1*)Nns^, *Rosa26^MerCreMer^*, MCM) have been described before (Turner et al., 1995; Schweighoffer et al., 2013). These were intercrossed to generate *Syk^fl/+^*MCM and *Syk^fl/−^*MCM strains. Gt(ROSA)26Sor^tm1(cre/ERT2)Thl^ (RCE) strain was also used for tamoxifen-inducible deletion of the BCR (Guo et al., 2007). Mice carrying a loxP-flanked rearranged VDJ_H_ gene in the IgH locus (*Igh^tm4Cgn^*, *Igh^B1-8f^*; Lam et al., 1997) and Tg(Emu-Bcl2l1)#Twb (Eμ-BclxL; Fang et al., 1996) were intercrossed with the MCM or RCE strains to generate *Igh^B1-8f/+^*Eμ-BclxLMCM or *Igh^B1-8f/+^*Eμ-BclxLRCE mice. Mice with a null allele of *Myd88* (*Myd88^tm1Aki^*, *Myd88^−^*; Adachi et al., 1998) were intercrossed with mice carrying the *Igh^B1-8f^* allele to generate *Myd88^+/+^Igh^B1-8f/+^* (*Myd88^+/+^*) and *Myd88^−/−^Igh^B1-8f/+^* (*Myd88^−/−^*) mice from which control and MYD88-deficient B cells could be isolated. For the in vivo experiments, TLR4-deficient (*Tlr4^tm1Aki/tm1Aki^*) animals were used as recipients (Hoshino et al., 1999). All strains were bred on a C57BL/6JNimr background (backcrossed at least eight times) within SPF facilities at the Francis Crick Institute, and in all studies mutant and control mice were littermates. All animal work was performed under the authority of a project license granted by the UK Home Office.

To generate control and SYK-deficient B cells, *Syk^fl/+^*MCM and *Syk^fl/−^*MCM mice at 8–12 wk of age were injected intraperitoneally for 5 d with 2 mg/d of tamoxifen (Sigma-Aldrich) in corn oil to induce Cre activity and delete the *Syk^fl^* allele. To generate B cells with and without BCR expression for assays of proliferation and up-regulation of activation markers, *Igh^B1-8f/+^*Eμ-BclxLMCM or *Igh^B1-8f/+^*Eμ-BclxLRCE mice at 8–12 wk of age were injected with tamoxifen as described above to induce Cre activity and delete the *Igh^B1-8f^* allele, and IgM^+^ and IgM^−^ cells were sorted by flow cytometry as described below. To generate B cells with and without BCR expression for immunoblotting analysis, they were purified by magnetic depletion (see below) from mice treated 10 d earlier with tamoxifen, using either *Igh^B1-8f/+^*Eμ-BclxL mice for IgM^+^ cells or *Igh^B1-8f/+^*Eμ-BclxLMCM or *Igh^B1-8f/+^*Eμ-BclxLRCE mice for IgM^−^ cells. In some instances, to generate larger numbers of B cells for immunoblotting experiments, radiation chimeras were generated using BM from the aforementioned mice to reconstitute RAG1-deficient mice (*Rag1^tm1Mom^*) that had been irradiated with 5 Gy as previously described (Schweighoffer et al., 2013).

### Generation of retroviral chimeras

Stocks of retroviruses were generated using pMIGR1 and MIGR1-Bcl-xL retroviral vectors (gift from W. Pear and D. Allman, University of Pennsylvania, Philadelphia, PA; Pear et al., 1998), as previously described (Schweighoffer et al., 2013). *Syk^fl/+^*MCM or *Syk^fl/−^*MCM mice were injected intraperitoneally with 100 mg/kg 5-fluorouracil (InvivoGen). 5 d later, BM was harvested, treated with ACK lysis buffer (155 mM NH_4_Cl, 10 mM KHCO_3_, and 100 µM EDTA), then cultured overnight in DMEM-plus (DMEM with 10% FCS [Lonza], 100 U/ml penicillin, 100 µg/ml streptomycin, 100 µM nonessential amino acids, 20 mM Hepes buffer, and 100 µM 2-mercaptoethanol) containing 100 ng/ml rmSCF (PeproTech), 6 ng/ml IL-3 (Sigma-Aldrich), and 10 ng/ml rmIL-6 (PeproTech). 24 h later, cells were moved onto retronectin-coated plates (Clontech T100B; Takara Bio Inc.), and viral supernatant was added and cultured overnight. This infection step was repeated the next day before cells were harvested and injected intravenously (at least 2.5 × 10^5^ cells/recipient) into RAG1-deficient recipient mice that had received 5 Gy irradiation. 6 wk after cell transplantation, the efficiency of infection was assessed by flow cytometry of peripheral blood, and tamoxifen treatment was started as described above.

### B cell purification by magnetic depletion

Splenic cell suspensions were treated with ACK lysis buffer to remove red blood cells and then stained with biotinylated anti-CD43 (ebioR2/60; eBioscience), anti-CD1d (1B1), anti-CD11b (M1/70; BioLegend), and anti-CD11c (N418; BioLegend) antibodies by standard procedures. To obtain IgM^−^ B cells, biotinylated goat anti–mouse IgM (SouthernBiotech) was also included. Magnetic Dynabeads M-280 Streptavidin (Thermo Fisher Scientific) were used to remove labeled cells according to the manufacturer’s instructions.

### Flow cytometry

For staining of cell surface molecules, ACK-treated cell suspensions were incubated in FACS buffer (PBS, 0.5% BSA, and 0.01% NaN_3_, pH 7.2–7.4) containing the appropriate, pretitered antibodies. Antibodies used are as follows, indicating antigen-fluorophore (clone): IgM-PECy7 (II/41), IgM-FITC (II/41), IgD-FITC (11-26), B220-PacBlue (RA3-6B2), CD5-PE (53-7.3), CD1d-FITC (1B1), F4/80-APC (BM8), CD11b-e450 (M1/70), TLR4-PE (MTS510), rat IgG2a isotype control (eBR2a; all from eBioscience); CD4-PerCP (RM4-5), CD8-PerCP (53-6.7), and CD19-PacBlue (6D5; all from BioLegend); CD86-PE (GL-1) and CD19-PerCP-Cy5 (1D3; both from BD); CD19-APC (RM705) and CD23-APC (MCD2305; Thermo Fisher Scientific); and CD4-PacificOrange (RM4-5) and CD8-PacificOrange (5H10; both from Invitrogen). Dead cells were excluded by staining with Zombie Aqua (BioLegend). For cell sorting, cells were stained with goat anti–mouse IgM Fab-FITC (Jackson ImmunoResearch Laboratories, Inc.), anti-CD23-PE (B3B4; BioLegend), anti-B220-BV421 (RA3-6B2; BioLegend), and anti-CD93-APC (AA4.1; eBioscience), along with PI (eBioscience) to exclude dead cells. Intracellular SYK expression was detected by fixing the cells for 30 min at 4°C with IC fixation buffer (eBioscience), followed by treatment for 3 min with 0.1% NP-40 (Sigma-Aldrich) and overnight incubation with anti-SYK (5F5; BioLegend) labeled with APC using the LYNX Rapid conjugation kit (Bio-Rad Laboratories). For intracellular IL-10 staining, cells were treated with Brefeldin A (1 µg/ml), ionomycin (0.5 µg/ml), PdBU (0.5 µg/ml), and LPS (10 µg/ml) for 5 h followed by cell fixation in 3.65% formaldehyde (Sigma-Aldrich), cell permeabilization in 0.1% NP-40 (Sigma-Aldrich), and staining with IL10-APC (JES5-16E3; BioLegend).

### B cell survival assay

Purified splenic B cells were cultured in full medium (DMEM, 10% FCS, penicillin/streptomycin, Hepes, nonessential amino acids, and 2-ME) in 96-well flat-bottom plates at a concentration of 2 × 10^6^ cells/ml. Stimulants used were as follows: anti-IgM F(ab′)_2_ (10 µg/ml; Jackson ImmunoResearch Laboratories, Inc.), LPS from *S. minnesota* R595 (10 µg/ml), lipid A from *S. minnesota* R595 (LAS; 10 µg/ml), lipid A from *E. coli* R515 (LAE; 10 µg/ml), synthetic monophosphoryl lipid A (SLA; 5 µg/ml; all from Enzo Life Sciences), CD40L (100 ng/ml, 1163-CL; R&D Systems), IL-4 (100 ng/ml; PeproTech), CpG (10 µg/ml; ODN 1826), Pam3CSK4 (100 ng/ml), and R848 (1 µg/ml; all three from InvivoGen). 48 h later, cells were harvested and live cells counted by flow cytometry using Calibrite beads (BD) and ToPro-3 (Thermo Fisher Scientific) as a live/dead marker. Live B cell numbers were normalized to input (taking purity values into account), then normalized to wild-type samples cultured in the presence of anti-IgM.

For studies with SYK inhibitors, the following inhibitors were included in cultures of wild-type B cells: PRT062607 (Selleckchem), R406 (Selleckchem), or BAY 61-3606 (EMD Millipore), using a twofold dilution series from 8 µM to 62.5 nM. As a control, B cells were cultured with a range of DMSO concentrations corresponding to those used in the inhibitor dilution series (0.08 to 0.0062%).

### Proliferation assay

Purified splenic B cells were labeled with eFluor 450 cell proliferation dye (CPD; eBioscience) according to the manufacturer’s protocol. Cells were cultured for 72 h in full medium in flat-bottom 96-well plates with stimulants as in cell survival assays, before harvesting and analysis on a flow cytometer. Dead cells were excluded by staining with Zombie Aqua (BioLegend). Calibrite beads allowed quantitation of live cells. The proliferation module of FlowJo was used to determine the percentage of cells that had divided at least once based on dilution of the CPD.

### In vivo proliferation assay

B cells were purified from female *Syk^fl/+^*MCM and *Syk^fl/−^*MCM mice treated 10 d earlier with tamoxifen. *Syk^fl/+^*MCM B cells were labeled with 2.5 µM CFSE (Invitrogen) in Dulbecco’s PBS for 7 min at 37°C, then incubated for 5 min more in full medium at 37°C. *Syk^fl/−^*MCM B cells were labeled with CPD eFluor450 (eBioscience) according to the manufacturer’s protocol. CFSE- and CPD-labeled cells were mixed in equal numbers, then 25–30 million cells were injected i.v. into female TLR4-deficient recipients, followed by i.v. injection of 10 µg LPS in 100 µl PBS. 3 d later, splenocytes were analyzed by flow cytometry. Proliferation of live (PI^−^) CD19^+^ cells labeled with CFSE or CPD was assessed by analysis of dye dilution using the proliferation module in FlowJo.

### Immunoblotting

B cells purified by magnetic depletion were rested at 37°C for 5 min (for pSYK or pAKT) or 30 min (for pERK and IκBα) before LPS (10 µg/ml), LAS (10 µg/ml), or CD40L (100 ng/ml) was added. Reactions were stopped by placing tubes on ice before centrifugation at 4°C, then cell pellets were snap frozen. Cell lysates were prepared using RIPA buffer: 50 mM Tris, pH 7.5, 150 mM NaCl, 2 mM EDTA, 1% Triton X-100, 5 mM dithiothreitol, 50 mM sodium fluoride, 1 mM sodium vanadate, 0.5% deoxycholate, 0.1% SDS, 2 mM sodium pyrophosphate, protease inhibitors (Roche complete mini protease inhibitor cocktail and PMSF), and PhosStop phosphatase inhibitor (Roche). After removal of cell debris by centrifugation, proteins in the cell lysates were separated on 10% SDS-PAGE gels and transferred onto Immobilon-FL PVDF membrane (EMD Millipore) by standard techniques. Membranes were blocked for 1 h in Odyssey Blocking Buffer (LI-COR Biosciences), and then probed with the following antibodies: anti–phospho-SYK/ZAP (Y352/Y319) rabbit polyclonal (#2701; Cell Signaling Technology); anti–phospho-ERK1/2 mouse monoclonal (sc-7383; Santa Cruz Biotechnology, Inc.); anti-ERK2 rabbit polyclonal (sc-154; Santa Cruz Biotechnology, Inc.); anti-SYK mouse monoclonal (clone 5F5; BioLegend); anti–phospho-AKT (S473) rabbit polyclonal (#9271; Cell Signaling Technology); and anti-IκBα (sc-371; Santa Cruz Biotechnology, Inc.). Staining was revealed using secondary antibodies: Alexa Fluor 680 goat anti–rabbit IgG (H+L; Thermo Fisher Scientific) and goat anti–mouse IRDye800CW (LI-COR Biosciences). Signals were detected with an Odyssey Infrared Imager (LI-COR Biosciences) and analyzed with the manufacturer’s software.

### Quantitation of mRNA

Total RNA was purified from B cells using TRIzol reagent (Invitrogen). RNA concentration was measured using a NanoDrop. Equal amounts of RNA (500 ng) were used to perform RT-PCR using Omniscript (QIAGEN). Primers used for the qPCR were as follows: Hprt (Mm03024075_m1), IL10 (Mm00439614_m1), IL6 (Mm00446190_m1), TNF (Mm00443258_m1), and cFos (Mm00487425_m1). mRNA levels for *Il6*, *Il10*, *TNF*, and *Fos* were normalized to Hprt.

### Cytokine secretion

Purified B cells were cultured with LPS (10 µg/ml) for 16 h. Concentration of secreted cytokines was measured from supernatants using mouse IL-10 Flex Set (BD) and mouse IL-6, TNFa FlowCytomix Simplex (Bender MedSystem).
