# Correction: TLR4 signals in B lymphocytes are transduced via the B cell antigen receptor and SYK

**DOI:** 10.1084/jem.2016111709292026c

**Published:** 2026-11-04

**Authors:** Edina Schweighoffer, Josquin Nys, Lesley Vanes, Nicholas Smithers, Victor L.J. Tybulewicz

Vol. 214, No. 5 | https://doi.org/10.1084/jem.20161117 | March 29, 2017

*JEM* regrets that, during the production process, an error was introduced in the second row of Fig. 4 B. The pSYK immunoblot in the first row was inadvertently copied, at a different exposure, into the SYK row below. The SYK row has been replaced with the correct immunoblot. In addition, the molecular weights of the LPS immunoblots in panel A have been corrected. The original and corrected figures are shown here. These corrections do not change the original conclusions of the article, and the figure legend remains unchanged.

The HTML and PDF versions of this article have been corrected. The errors remain only in PDFs downloaded before October 27, 2026.

**Figure:**
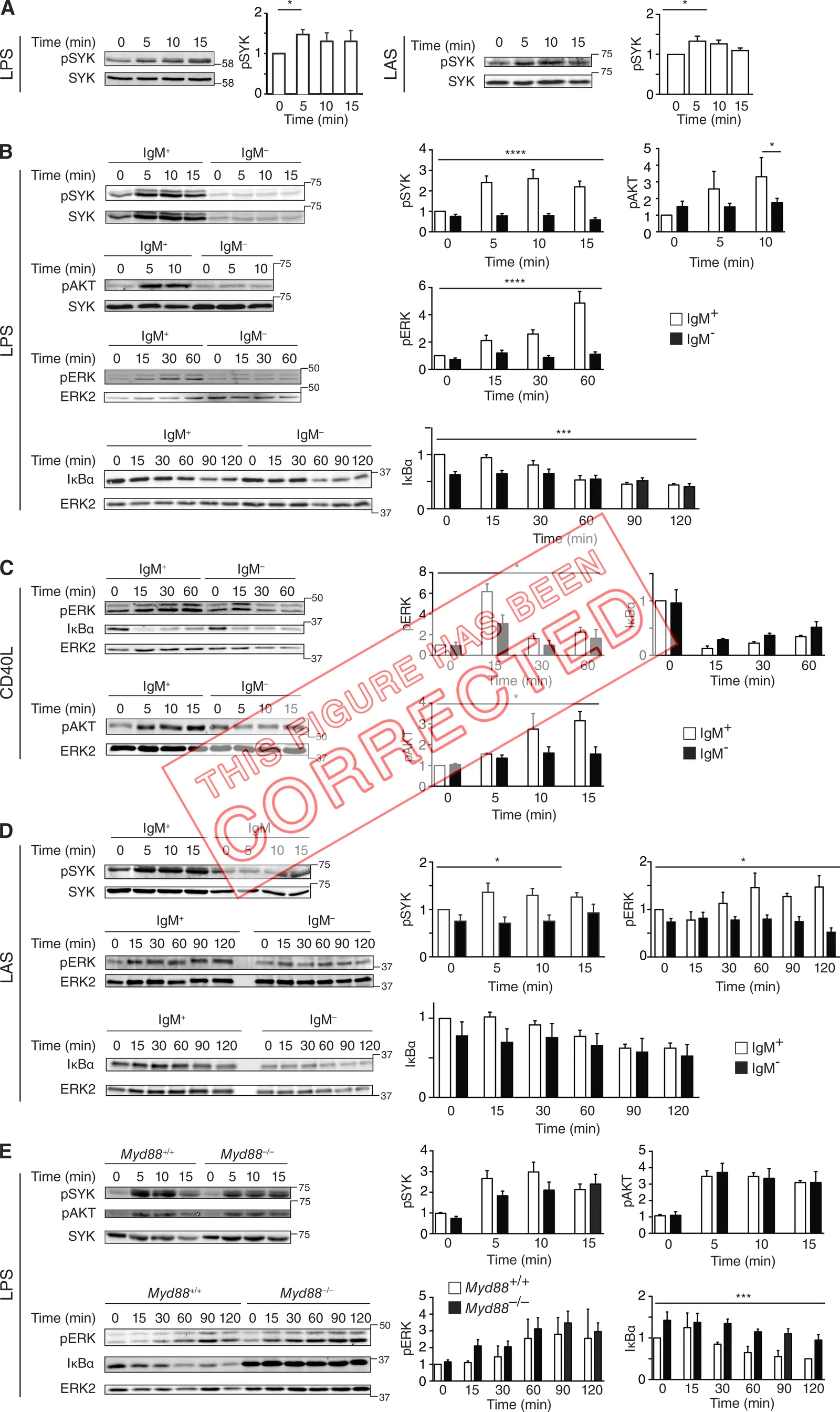


**Figure 4.**
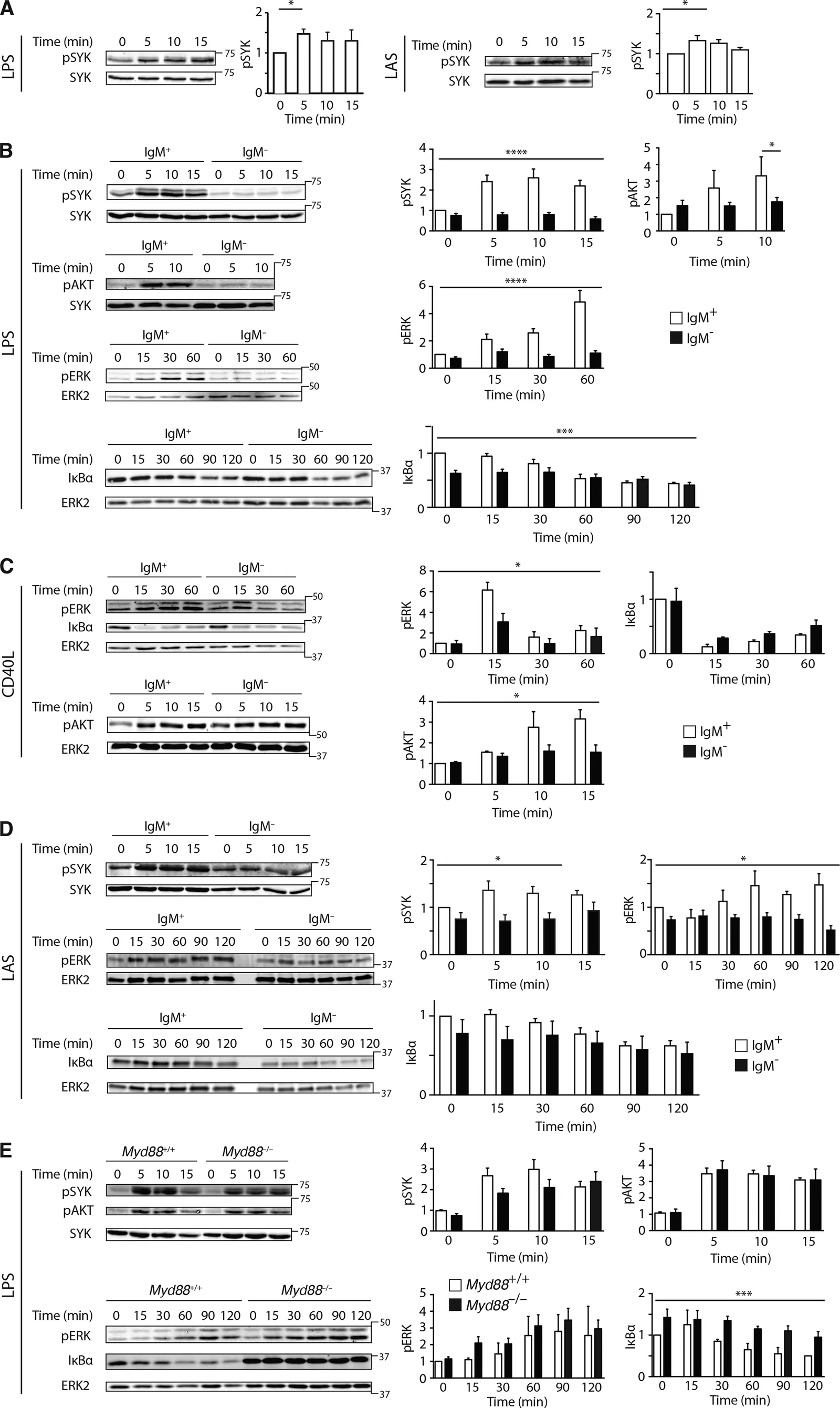
**BCR is required for TLR4-induced activation of SYK. (A)** Immunoblot of lysates from WT B cells stimulated for various times with LPS (left) or LAS (right). Blots were probed with antibodies to pSYK and SYK. Graphs show mean ± SEM levels of pSYK normalized to SYK or tubulin and then to unstimulated cells (time 0), quantitated from multiple immunoblots. *n* = 4 (LPS) and 3 (LAS). **(B–E)** Immunoblots of lysates from IgM^+^ or IgM^−^ B cells (B–D) or *Myd88*^*+/+*^ or *Myd88*^*−/−*^ B cells (E) stimulated with LPS (B and E), CD40L (C), or LAS (D) for the indicated times. Blots were probed with antibodies to pSYK, pAKT, pERK, SYK, ERK2, and IκBα. Graphs show mean ± SEM levels of pSYK, pAKT, pERK, and IκBα normalized to SYK or ERK2 and then to the levels in unstimulated control B cells (time 0), quantitated from multiple immunoblots. Minimum number of repeats per time point: B, 9 (pSYK except 3 for 15 min), 8 (pAKT), 7 (pERK), and 5 (IκBα); C, 3 (pERK), 3 (IκBα), and 2 (pAKT); D, 3 (pSYK), 4 (pERK), and 4 (IκBα); E, 9 (pSYK) and 7 (pAKT); 2 (pERK and IκBα in WT), 4 (pERK and IκBα in *Myd88*^*−/−*^). Numbers on the right-hand edge of all blots indicate molecular mass makers (kilodaltons). Statistical analysis was performed using a Mann–Whitney test (A and B, pAKT) or two-way ANOVA (all other graphs; p values for effect of genotype): *, P < 0.05; ***, P < 0.001; ****, P < 0.0001.

